# GMP manufacturing of umbilical cord blood mesenchymal stem cell-released exosomes and verification of wound healing efficacy

**DOI:** 10.7150/ntno.105433

**Published:** 2025-03-03

**Authors:** Jae-Yong Lee, Jaehyoung Kim, Kyuheum Na, Hee-Jin Ahn

**Affiliations:** Cytotherapy R&D Center, Primoris Therapeutics Co., Ltd., #1504, A Bldg., 60 Haahn-ro, Gwangmyeong-si, Gyeonggi-do 14332, South Korea.

**Keywords:** human umbilical cord blood mesenchymal stem cells, exosomes, GMP manufacturing, tissue regeneration, wound healing

## Abstract

**Rationale:** Drug development research using exosomes is being actively conducted worldwide. However, clear management standards and verification systems for the use of exosomes as drugs are still being developed. In this study, the effectiveness of exosomes as drug candidates was verified after production in accordance with the guidelines for donor suitability, cell bank construction of cell therapy drugs, and quality control of extracellular vesicles published by the Korean Ministry of Food and Drug Safety (KMFDS).

**Methods:** Basic characteristics were identified using umbilical cord blood mesenchymal stem cells (UCBMSCs)-released exosomes produced in accordance with the three guidelines, and internal component analysis of proteins, lipids, and nucleic acids was performed. In addition, various types of *in vitro* and *in vivo* experiments confirmed the skin tissue regeneration efficacy of exosomes.

**Results:** In addition, treatment of two types of skin cells (dermal fibroblast and keratinocyte) with exosomes resulted in a statistically significant increase in the proliferation and migration of skin cells and inhibition of the secretion of pro-inflammatory substances in an inflammatory environment. In an animal model of wound injury, exosome treatment accelerated the wound healing process. These* in vitro* and *in vivo* experiments confirm that UCBMSCs-released exosomes have tissue regeneration and inflammation suppression properties.

**Conclusions:** This study presents the processes and quality control items that can produce exosomes as drugs in good manufacturing practice facilities and shows the possibility of developing drugs for various diseases based on the inherent efficacy of UCBMSCs-released exosomes. This study is the first to show how exosomes can be produced and quality-validated in a GMP facility for use as drugs, which will accelerate the time to market for future exosomes base bio-new drug.

## Introduction

Extracellular vesicles are collectively referred to as vesicles of various sizes secreted by cells, but in the biopharmaceutical area, they usually refer to exosomes with a diameter of 30 to 150 nm. Exosomes are also described as "avatar of cells" because they are made and released containing various substances in the cytoplasm by the intrinsic production process in the cell and reflect the characteristics of mother cells **1**. Compared to the average cell size of 10 to 30 μm in diameter, the size of the exosome (30 to 150 nm) is only up to 1/1,000 on a cell basis. The size of this exosome is considered to be the biggest advantage in that it can pass intravenous injection and brain-blood barrier, exceeding the limitations of stem cell treatments that only local injection was possible. Currently, the development of treatments for various diseases using exosome is actively underway around the world, but no cases have been approved yet **2-4**.

Stem Cells has the effect of healing damaged tissues based on its own self-renewal and differentiation potency, so drugs for various diseases have been released, and many drugs are still being developed. Stem cells used as general treatments are adult stem cells, which are classified into bone marrow, adipose, and umbilical cord blood mesenchymal stem cells **(UCBMSCs)** according to the tissue that can secure stem cells. Among the three adult stem cells, UCBMSCs have better cell growth than other tissue derived stem cells, so it is possible to establish a master cell bank **(MCB)** using one lot of stem cells secured from one donor. It is also well known to have the best efficacy as a pharmaceutical raw material among adult stem cells by containing or secreting a lot of various inflammation inhibition and tissue regeneration proteins **5-7**. However, due to the technical limitations of isolating and culturing very few stem cells in the umbilical cord blood **(UCB)**, there is only one actual drug released, and CARTISTEM^®^, an osteoarthritis treatment by MEDIPOST Co., Ltd. is the only case **8**. In this study, we secured our own technology for isolating and mass-culturing stem cells from UCB and used this technology to establish a mesenchymal stem cell MCB derived from allogeneic cord blood. Based on these origin materials, we intend to develop medicines using stem cell released exosomes.

In order to use biopharmaceuticals using human cells or tissue-derived substances as medicines, production and quality management at good manufacturing practice **(GMP)** facilities are essential **9-11**. Currently, clinical trials of exosomes as drugs are underway around the world, targeting a variety of diseases, but none have yet been licensed and marketed, so the process of producing and validating exosomes as drugs needs to be newly established. In this study, various experiments were conducted in accordance with the processes and standards that can be performed at GMP facilities, from securing and verifying the safety of origin materials through donor donation to selecting and characterizing cell lines for exosome releasing cells. Using the exosomes secured in this way, wounds that require inflammation suppression and tissue regeneration processes were selected as diseases and the efficacy and mechanism were verified.

## Methods

### Securing of UCB

UCB was provided from FORMIZ Women's Hospital for UCBMSCs isolation (IRB No. P01-2020-31-005). At least 80 ml of UCB and the mother's blood for donor eligibility determination tests were collected and secured under the guidance of the medical staff when the mother who agreed to give birth in advance.

### Securing umbilical cord blood and isolating and culturing UCBMSCs

UCBMSCs were obtained from healthy pregnant women with their consent. The cord blood was mixed with a buffer solution in a 1:1 ratio and then mixed with Ficoll-plaque (Cat. no. GE17-1440-02, Cytiva, MA, USA) solution in a 4:3 ratio (Blood Sample 4: Ficoll-Plaque 3). The mixture was centrifuged at 400g for 30 minutes, and the mononuclear cell layer was separated. The separated mononuclear cell layer was washed three times with PBS (Cat. no. SH30256.01, Cytiva, MA, USA) and then resuspended in a culture medium. The cells were divided into 6-well plates and incubated at 37°C in a 5% CO2 incubator. After 72 hours, the suspended cells were removed by washing with PBS, and only the cells attached to the plate were incubated. After colonies formed from the attached and cultured cells, only those cells were selected and cultured on a new plate for further experiment.

### Identification of UCBMSCs

The cultured cells were detached with TryPLE (Cat. no. 12604013, Thermo Fisher Scientific, MA, USA), then inactivated with a culture medium (Cat. no. K3901, Kangstem biotech, Seoul, Korea) before harvesting the cells by centrifugation. The harvested cells were washed with PBS and resuspended in 1 ml of PBS containing 0.5% FBS (Cat. no 10082147, Thermo Fisher Scientific, MA, USA). CD44 (Cat. no 11-0441-82, Invitrogen, CA, USA), CD73 (Cat. no. 11-0739-42, Invitrogen, CA, USA), CD105 (Cat. no. 17-1057-42, CA, USA), CD11b (Cat. no. 11-0118-42, CA, USA), CD45 (Cat. no. 11-0459-52 CA, USA), CD19 (Cat. no. 11-0199-42, CA, USA), CD34 (Cat. no. 11-0349-42, CA, USA) HLA-DR (Cat. no. 11-9956-42, CA, USA), fluorescently conjugated antibodies were added and incubated at room temperature for 1 hour. The cells were then washed twice with PBS containing 0.5% FBS and analyzed. The cell analysis was performed using Cytoflex (Beckman Coulter, CA, USA) and analyzed using CytExpert (Beckman Coulter, CA, USA) software.

### Inducing of differentiation to 3 types cells using UCBMSCs

UCBMSCs (passage 6, 3x10^5^ cells) were seeded onto a 6-well plate and cultured at 37°C with 5% CO2 in an incubator. To induce adipogenic differentiation, UCBMSCs were cultured in Adipogenesis Differentiation Medium (Cat. no. A1007001, Thermo Fisher Scientific, MA, USA) for 14 days. The differentiated cells were fixed with 4% paraformaldehyde and stained with Oil-Red O solution for 15 minutes. For osteogenic differentiation, UCBMSCs were cultured in Osteogenesis Differentiation Medium (Cat. no. A1007201, Thermo Fisher Scientific, MA, USA) for 14 days. The differentiated cells were fixed with 4% paraformaldehyde and stained with Alizarin Red solution for 15 minutes. For chondrogenic differentiation, UCBMSCs were cultured in Chondrogenesis Differentiation Medium (Cat. no. A1007101, Thermo Fisher Scientific, MA, USA). When the cells formed a pellet during culture, they were transferred to a 15 mL tube and cultured for 21 days. After differentiation, the cells were fixed in 4% paraformaldehyde, cryosectioned, and stained with Alcian Blue to detect cartilage formation.

### mRNA isolation, cDNA synthesis, quantitative RT-PCR

Total RNA was isolated using the PureLink™ RNA Mini Kit (Cat. no.: 12183020, Invitrogen, CA, USA) and quantified with a nanodrop (Thermo Fisher Scientific, MA, USA). 1 μg of Total RNA was then reverse transcribed into cDNA using AccuPower® RT PreMix & Master Mix (Cat. no. K-2041-B, Bioneer, Daejeon, Korea) following the manufacturer's protocol. qRT-PCR was performed using a QuantStudio™ 3 Real-Time PCR System (Applied Biosystems™, CA, USA) with SyBR Premix (Cat. no A25779, Thermo Fisher Scientific, MA, USA) and primers listed in **Table 5**. The expression levels were normalized to the housekeeping gene GAPDH, and the relative gene expression was calculated using the 2-ΔΔCT method. **Table 5** shows the primer sequence for the genes used in RT-PCR.

### Enzyme-linked immunosorbent assay (ELISA)

The levels of each cytokine, including TNF-α (Cat. no. DTA00D, R&D System, NE, USA), IFN-γ (Cat. No. DIF50C, R&D System, NE, USA), IL-1β (Cat. no. DLB50, R&D System, NE, USA) were detected and quantified by immunoassay according to the manufacturer's manual. All experiments were performed in triplicate.

### Western Blot

The following antibodies were used in this study: Anti-Fibronectin antibody (Cat. No. ab2413, Abcam, Cambridge, UK), Akt Antibody (Cat. No. 9272S, Cell Signaling, MA, USA), Phospho-Akt (Cat. no. 4060S, Cell Signaling, MA, USA), Phospho-p38 MAPK (Cat. no. 4511S, Cell Signaling, MA, USA), p38 MAPK (Cat. no 8690S, Cell Signaling, MA, USA), Phospho-SAPK/JNK (Cat. no. 4668S, Cell Signaling, MA, USA), SAPK/JNK (Cat. no. 9252S, Cell Signaling, MA, USA), β-Actin, (Cat. no. 4967S, Cell Signaling, MA, USA), Anti-Rabbit IgG, (Cat. no. 7074S, Cell Signaling, MA, USA) and then lysed in tissue lysis buffer containing a protease inhibitor. The lysates were centrifuged and the supernatant was collected and quantified by the BCA method for protein quantification before using for analysis. The samples were analyzed by sodium dodecyl sulfate-polyacrylamide gel electrophoresis, followed by Semi-Dry Transfer to a PVDF membrane. The membrane was blocked with 5% BSA and then incubated with the primary antibody overnight at 4°C. After washing with 1X TBST, the membrane was incubated with the secondary antibody at room temperature for 1 hour and then washed again with 1X TBST. Detection was performed using a RAS machine.

### Securing of UCBMSC released exosomes

In order to secure UCBMSCs-releasing exosomes (ExoPlus^TM^), cells were thawed and mass-cultured from MCB. Since then, cell proliferation does not occur any more, and it is cultured for a certain period of time in a serum-free medium that promotes the releasing of exosomes from the stem cell, thereby inducing a sufficient amount of exosomes to be contained in the conditioned medium. A total of three steps were constructed to isolation and purification for high-purity exosomes from the secured conditioned medium. As the first step, substances in the culture medium smaller than exosomes were removed through a Tangential Flow Filtration (TFF) process. Subsequently, in the second step using centrifugation, a heavier substance (debris) was removed through a centrifugation process of 3,000 g, and a lighter substance was removed through a high-speed centrifugation process of 100,000 g. In the last third step, to remove substances smaller than exosomes, Size Exclusion Chromatography was performed to recover only exosomes of a specific fragment, thereby producing high purity exosomes (ExoPlus^TM^).

### FBS-free testing of UCBMSCs exosome production at every step of the process

To verify the residual presence of FBS used in the cell culture process during exosome production, we collected samples at each process step and analyzed them using a Bovine Albumin ELISA kit (Cat No. 8100, Alpha Diagnostic International, TX, USA). Each sample was mixed with 1X Sample Dilution Buffer and diluted appropriately for the respective samples, and the analysis was performed in duplicate.

### Confirm the size distribution of exosomes_nanoparticle tracking analysis (NTA) measurement

Exosomes were diluted in PBS (Cat. no. SH30256.01, Cytiva, MA, USA) to a final volume of 1 ml and measured. All measurements were performed by diluting with PBS to the optimal dilution value, determined to achieve ideal particle values per frame (20-80 particles/frame). The settings were configured according to the manufacturer's manual and the engineer's recommendation. Exosome measurements were taken at 25°C for 60 seconds, 5 times with a camera level of 12-15, and manual focus adjustment.

### Observation of morphology of exosomes_Cryo-transmission electron microscope (TEM)

The TEM and Cryo-TEM images were obtained by commissioning the Shared Research Facilities of the College of Agriculture and Life Sciences at Seoul National University. TEM measurements were performed by loading 5 μl of concentrated exosome samples purified from UCBMSCs conditioned media onto glow-discharged carbon-coated copper grids using Amicon Ultra-15 (Cat. no. UFC900324, Merck, NJ, USA). The grids were allowed to react for 1 minute and then washed with D.W before being stained in 2% (w/v) uranyl acetate solution for 1 minute to perform negative staining. The samples were then air-dried and measured using TEM (Talos L120C, FEI, Czech) at 120 kV. Cryo-TEM measurements were performed by loading exosome samples onto glow-discharged C-flat holey carbon grids and freezing them in liquid ethane. The samples were then measured using TEM (Talos L120C, FEI, Czech) at 120 kV.

### Analysis of exosomes markers using a flow cytometer

The exosomes harvested from the conditioned medium of UCBMSCs were incubated with Latex Beads (Cat. no. A37304, Thermo Fisher Scientific, MA, USA) at 4°C overnight. The exosomes bound to Latex Beads were washed with PBS and then resuspended in 1 ml of 0.1% BSA in PBS Buffer. The exosomes were then incubated with antibodies for each exosome marker (CD9, CD63, CD81) at room temperature for 1 hour. After incubation with antibodies, the exosomes were washed with 0.1% BSA in PBS buffer and analyzed. The analysis was performed using Cytoflex (Beckman Coulter, CA, USA) and analyzed using CytExpert (Beckman Coulter, CA, USA) software.

### Skin cell culture and proliferation assay

The Human dermal fibroblast (HDF, Cat. No PCS-201-012, VA, USA) and Human keratinocyte (HKC, Cat. No PCS-200-011, VA, USA) cell lines used in the experiment were purchased from ATCC (Manassas, VA, USA). HDF cells were cultured in a DMEM (Cat. No 11965092, Thermo Fisher Scientific, MA, USA) medium containing 10% FBS (Cat. no. 10082147, Thermo Fisher Scientific, MA, USA) at 37°C in a 5% CO2 incubator. HKC cells were cultured in RPMI-1640 (Cat. No 11875093, Thermo Fisher Scientific, MA, USA) medium containing 10% FBS at 37°C in a 5% CO2 incubator. To analyze cell proliferation, each cell type was seeded at a density of 10,000 cells/cm^2^ in 6-well plates and cultured for one day. They were then treated with their respective experimental and control groups. After 24 or 48 hours, cells were harvested, and cell counts were measured. Additionally, cell proliferation was assessed using the CCK-8 Assay (Cat. No CK04, Kumamoto, Japan) as an alternative method.

### Cell migration assay

To assess the migratory capacity of HDF cells and HKC cells, Transwell chamber (Cat. no. 3422, Corning, AZ, USA) were used. After culturing each cell, they were harvested and resuspended in a serum-free medium at a concentration of 1x10^5^ cells/ml. Subsequently, 100 μl of each cell suspension was loaded onto the upper chamber of the Transwell. The cells were then incubated for one day at 37°C in a 5% CO2 incubator. To the upper chamber of the Transwell, control groups (PBS) and ExoPlus^TM^ were added, with each receiving 50 μl. The lower chamber was loaded with a culture medium containing 10% FBS (Cat. no. 10082147, Thermo Fisher Scientific, MA, USA). After 48 hours of incubation at 37°C in a 5% CO_2_ condition, cells on the upper side of the filter were removed with a cotton swab. Cells that had migrated to the lower side of the insert were fixed with 4% paraformaldehyde (Cat. no. PC2031-100-00, Biosesang, Gyonggi-do, Korea) for 10 minutes, stained with crystal violet (Cat. no. V5265, Merck, NJ, USA) for 20 minutes, and then washed with PBS. Subsequently, they were observed under a microscope.

### *In vivo* assay using wound animal model

We purchased Sprague Dawley Rat at 7 weeks of age from Orient Bio Inc. All mice were acclimatized under optimal breeding conditions for one week before the experiments were conducted. All procedures were reviewed and approved by the Seoul National University Institutional Animal Care and Use Committee. The rats were anesthetized with isoflurane (Hana Pharm, Gyeonggi-do, Korea) and their fur was removed. Biopsy Punch (Acuderm, FL, USA) was used to induce wounds. To prevent skin contraction after wound induction, the skin was secured with rubber rings. Subsequently, control groups (PBS) and ExoPlus^TM^ were applied, with each receiving 100 ul. The wound area was then covered with Tegaderm (3M, MN, USA) and dressed with a compression bandage. After 7 days, the extent of wound healing was assessed, and the induced wound area was isolated. It was then fixed in 4% formaldehyde (Biosesang, Gyeonggi-do, Korea) and sent to T&P Bio for H&E staining and Masson's trichrome stain.

### Statistical analysis

All the data were shown as meaning ± standard deviation (SD). The statistically significant differences between groups were assessed by t-test using GraphPad Prism 5 software. P values < 0.05 were considered significant. *P < 0.05, **P < 0.01. The values are shown in the figures.

## Results

### Eligibility determination for donors

Donor eligibility determination tests were performed on two samples, the mother's blood and cord blood, based on the contents specified in the "Guideline on Eligibility Determination for Donors of Cell Therapy Products. 2015.05" published by the Ministry of Food and Drug Safety of Korea (KMFDS) **12**. Based on the guidelines, stem cells were isolated using UCB that was confirmed to be negative in all applicable test items **(Table 1)**.

### Isolation and characterization of UCBMSCs

**Figure 1:**
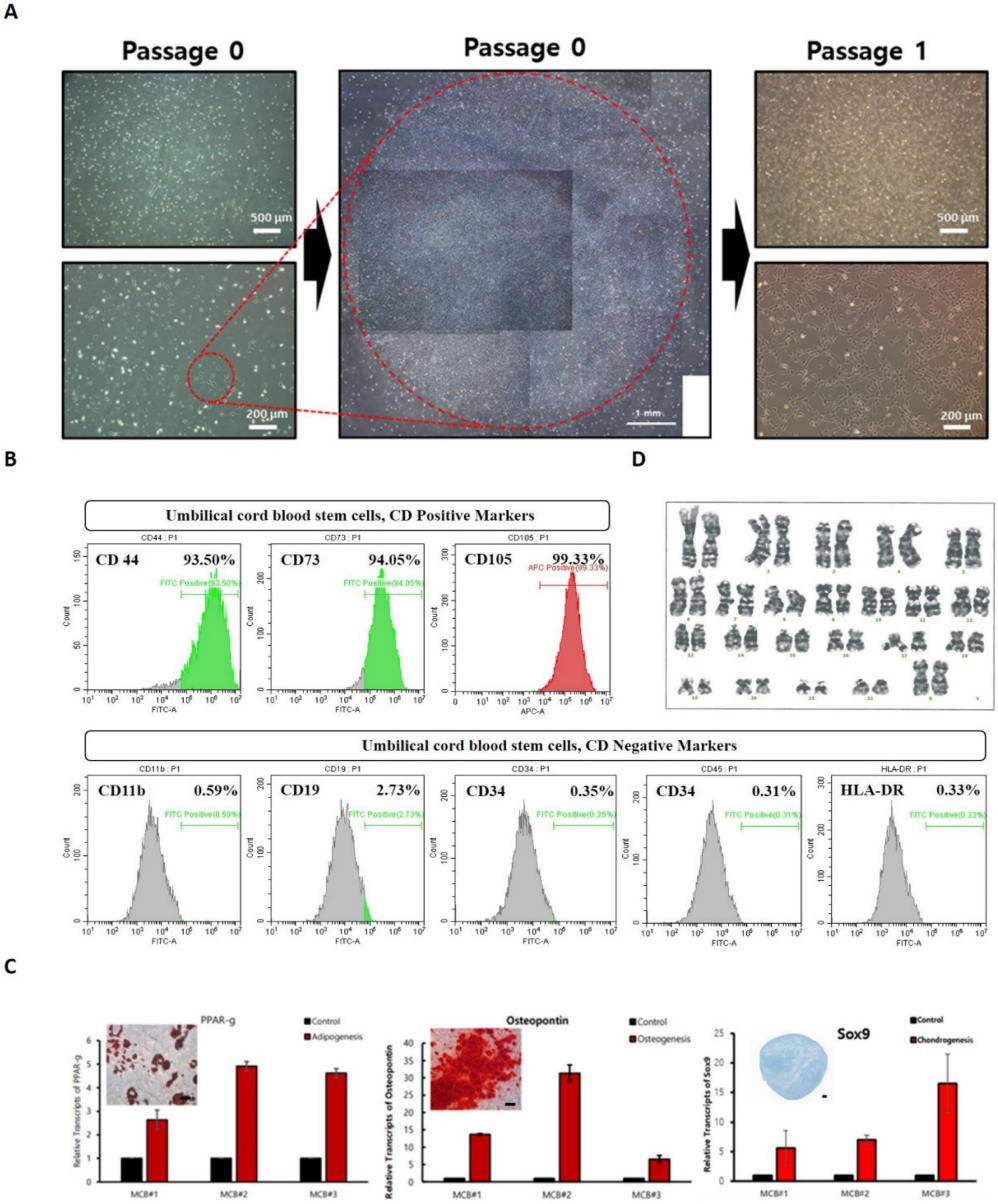
Characterization of human UCBMSCs. **(A)** The morphology of cells after the formed of colony from sing stem cells and one time passaging. **(B)** The cytometry analysis for positive surface markers of UCBMSCs and negative markers. **(C)** Analysis of related gene and protein expression after differentiation into three types of cells (Adipogenesis (PPAR-), Osteogenesis (Osteopontin) and Chondrogenesis (Sox9) using isolated UCBMSCs. The scale bar in the photo image is 200 ㎛. **(D)** Confirmation of genetic stability of UCBMSCs by analysis of karyotype.

In the process of culturing mononuclear cells in cord blood, floating cells were removed through the culture media change process, and stem cells were isolated by observing the growth patterns of cells growing attached to the bottom of a culture dish. Stem cells have the characteristics of growing short and sharped cells stably attached to the culture dish bottom, and the initial cell proliferation time is short within 24 hours, forming cell colonies within days **(Figure 1A)**. The colony was made into single cells using cell dissociation enzyme and then cultured in a new culture dish to induce cell proliferation and then a test was performed to analyze UCBMSCs characteristics. Cell surface protein analysis was performed using flow cytometer to identify whether UCBMSCs were correct, confirming that the expression of five negative markers (CD11b, CD19, CD34, CD45, and HLA-DR) was less than 1%, and that three positive markers (CD44, CD73, and CD105) were more than 90% **(Figure 1B)**. To confirm the differentiation potency, one of the characteristics of stem cells, Adipogenesis, Osteogenesis, and Chondrogenesis were induced to confirm the expression of representative genes for each tissue and changes in cell characteristics through staining **(Figure 1C)**. The karyotype analysis was performed to determine whether UCBMSCs secured after identification of UCBMSCs maintain genetic stability during the isolate and culture process, and 46 chromosomes were confirmed to be normal **(Figure 1D)**. In this analysis, at least 20 cells per passage were analyzed using three passages to increase accuracy (Suppl Figure 1). These results show that cells isolated and cultured in UCB are stem cells with normal characteristics.

### Establishment and character analysis of MCB

**Figure 2:**
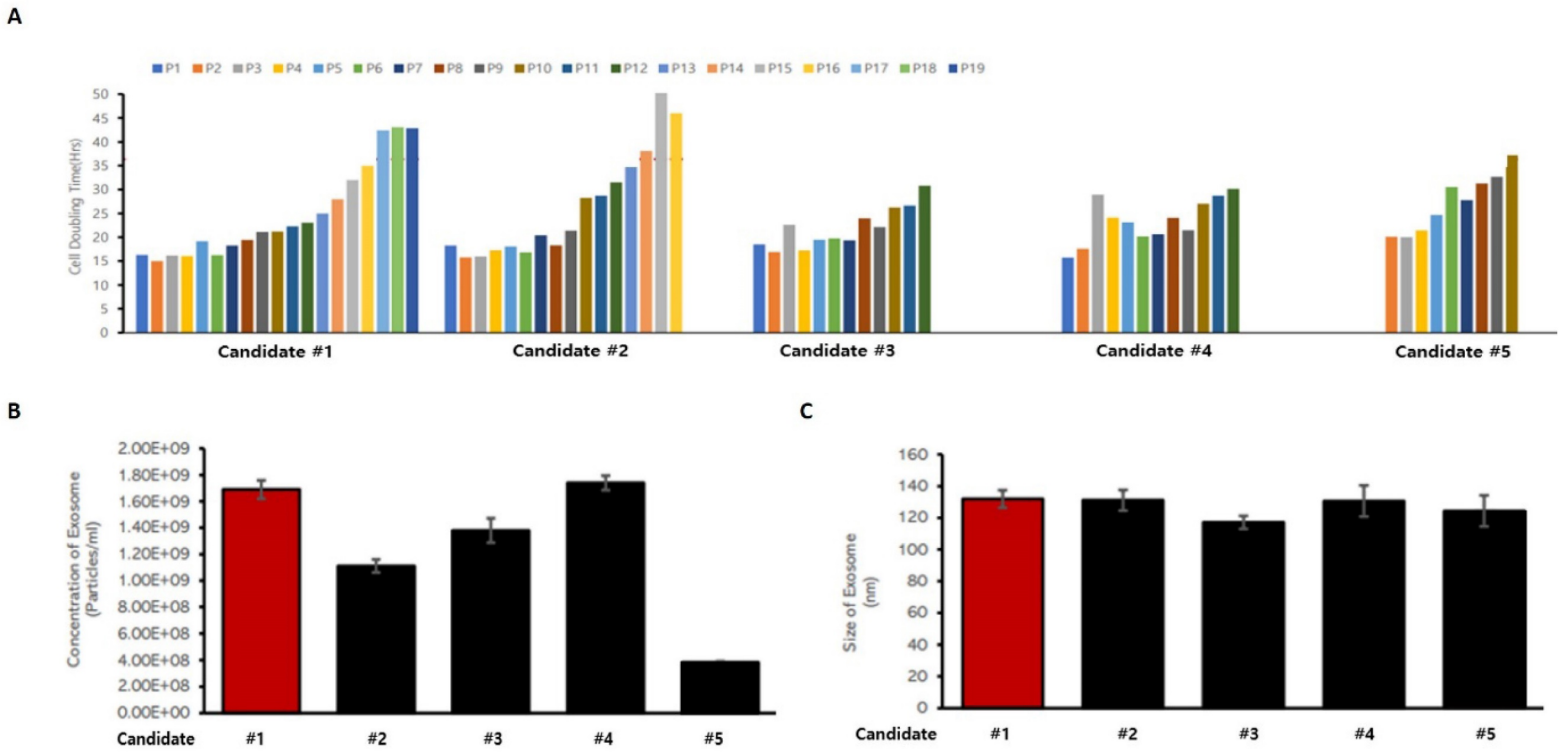
Criteria for screening UCBMSCs lines for MCB construction. **(A)** Analysis of cell proliferation time and culture potential for each candidate cell lines. **(B)** Analysis of the particle numbers of exosomes released from candidate cell lines. **(C)** Analysis of the size of exosomes released from candidate cell lines.

When biomedicines are manufactured using living cells secured from donors, effective quality control is possible only when the origin and source of the cells are clarified, and cell banks are established and characterized. To this end, characteristics between cell lines were compared and analyzed to construct a MCB using a specific cell line among a number of UCBMSCs. In order to select MCB cell stocks, three items were analyzed: cell proliferation ability, including stem cell proliferation time, exosome releasing rate under the same culture conditions, and the size of the released exosome. An analysis was performed using a total of five UCBMSCs lines to select one cell line. The cell line was able to be passage for the longest time and had a short proliferation time for passaging among the five cell lines **(Figure 2A)**. This means that the largest number of UCBMSCs can be secured when cultured for the same time. In addition, as a result of checking the number of exosomes released in the process of cultivating stem cells, it was confirmed that the second largest number of exosomes was released in the candidate cell line, and the difference from the most released cell line was insignificant **(Figure 2B)**. When analyzing the size of exosomes secreted by five cell lines, it was analyzed evenly with an average diameter of about 130 nm without any cell-week deviation **(Figure 2C)**. Based on the analysis results of these three characteristics, candidate #1 cell line was selected as a candidate for cell line for the construction of an UCBMSCs MCB. Finally, characteristic analysis was performed on the test items presented in the "Guidelines for Cell Bank Evaluation of Cell Therapy Products, 2021.08" published by the KMFDS **13** to select cell line for MCB. A total of 13 tests were conducted to confirm suitability and finally selected as MCB **(Table 2)**.

### Identification of UCBMSC released exosomes

**Figure 3:**
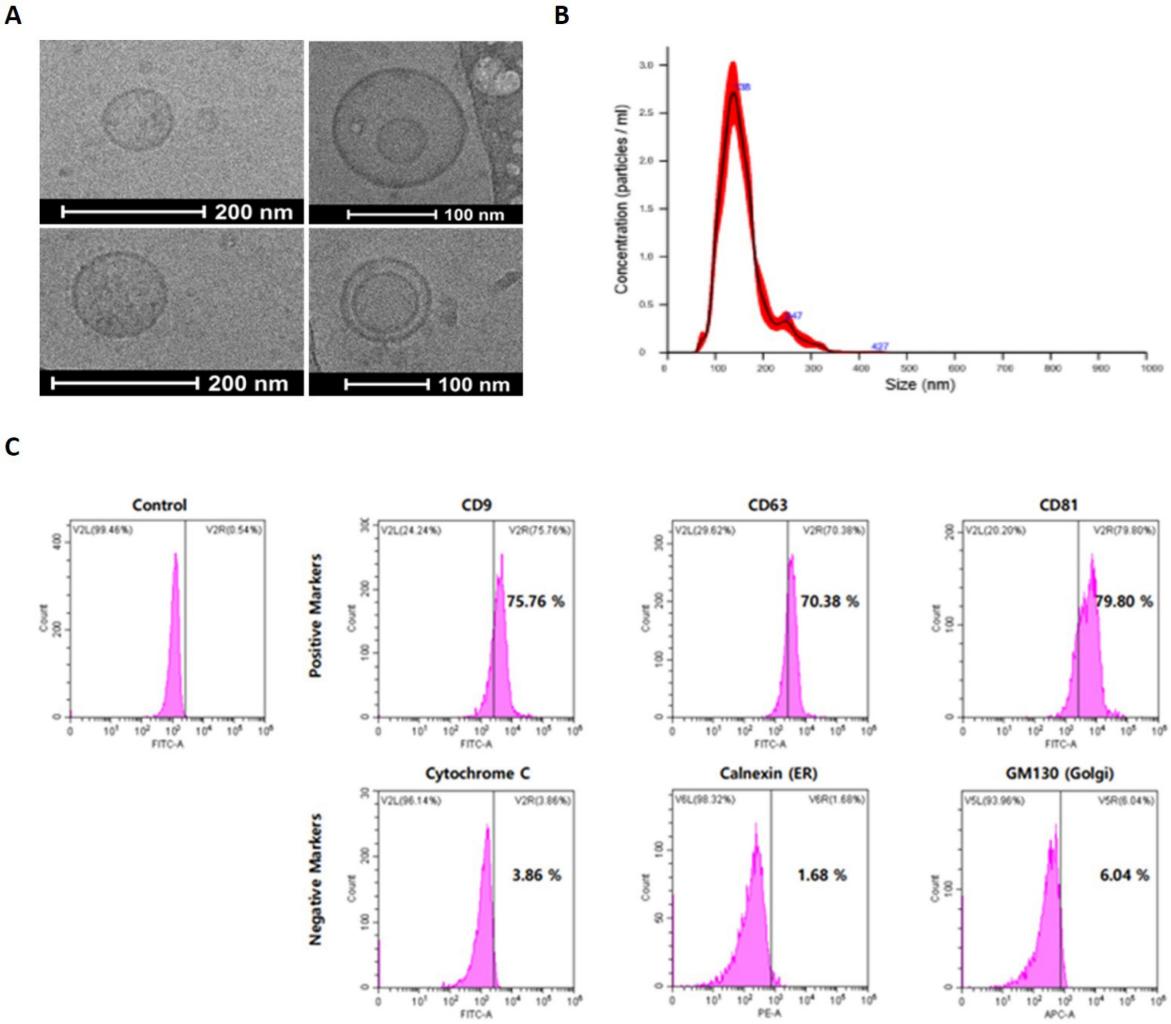
Identification and characterization of exosome from UCBMSCs. **(A)** The morphology observation of exosomes using Cryo-TEM. **(B)** Analysis of the size distribution of exosomes. **(C)** Identification of exosomes-specific surface markers (CD9, CD63 and CD81_positive markers) and intracellular organ markers (Cytochrome C, Calnexin and GM130_negative markers).

An identification experiment was first performed to analyze the characteristic of exosomes that went through the three-step isolation and purification process. To analyze the morphological characteristics of exosomes, Cryo-TEM was used to photograph the appearance, and as a result, a circular vesicle-type exosome was observed **(Figure 3A)**. In addition, as a result of analyzing the distribution of exosomes by diameter through NTA, exosomes with an average diameter of 110 nm were identified **(Figure 3B)**. For exosome identification, expression of exosome-specific membrane proteins was confirmed by FACS analysis. Through FACS analysis, it was confirmed that CD9, CD63, and CD81, which are unique surface markers of UCBMSCs-releasing exosomes, were expressed more than 70% higher. In addition, Cytochrome C (Mitochondria protein), Calnexin (ER protein), and GM130 (Golgi protein), which are proteins in small organs in cells with lipid bilayer structures similar to exosomes, were analyzed to be less than 10% **(Figure 3C)**. In addition, during the process of isolating/purifying exosomes, it was confirmed whether other organelles present in the conditioned media were included. Through RT-PCR analysis, mRNA expression of cytochrome C (mitochondria), ERP29(endoplasmic reticulum), GOLM1 (Golgi), and NUP153 (Nuclear) was confirmed. As a result of the analysis, it was confirmed that four types of organelles-related genes were not expressed (**Suppl. Figure 3**). Through these results, identification and purity of exosomes secured through the three-step isolation and purification process were confirmed.

### Exosome internal component analysis

Due to the nature of the intrinsic formation process, exosomes are formed containing the intracellular substrate of the mother cell and released out of the cell. According to “Guideline on Quality, Non-clinical and Clinical Assessment of Extracellular Vesicles Therapy Products 2018.12" of KMFDS **14**, it is suggested that protein, lipids, and nucleic acid components among the internal components of exosomes need to be analyzed, which is an important indicator of the quality control of exosomes. Among the various components present inside the exosome, three representative substances (protein, lipid and nucleic acid) were requested to an external institution for analysis. Protein and lipid components were analyzed by new drug development support center of OSONG Medical Innovation Foundation (in South Korea), and nucleic acid components were analyzed by requesting ROKIT Genomics Inc. (in South Korea). For the analysis of the components in exosomes, three lots of exosomes were independently produced and each was used as a sample.

**Figure 4:**
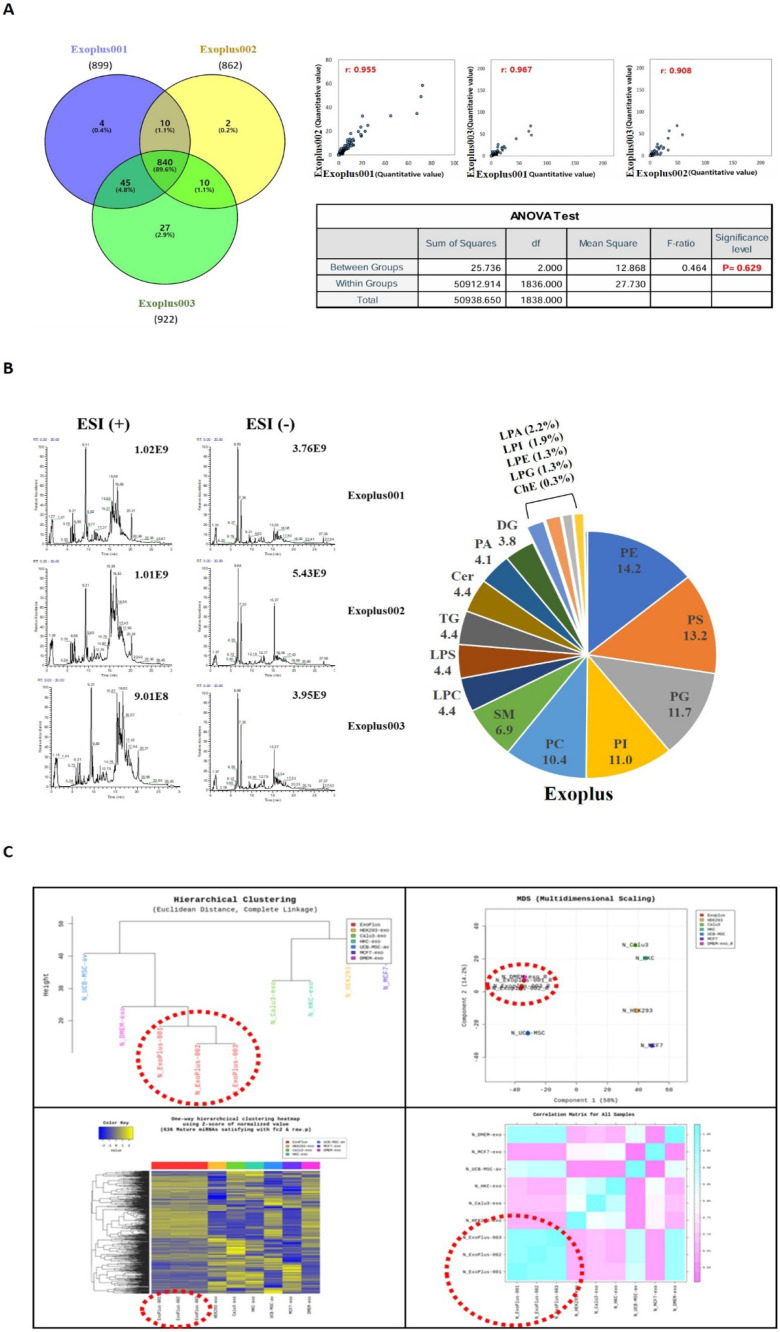
Analysis of ingredients in exosomes using independent 3 lots of exosomes. **(A)** Identifying the number of proteins commonly analyzed between the three lots and the similarity between lots. **(B)** Identification of similarity in the expression patterns of key lipid components between the three lots **(C)** Identification of similarity between exosomes of 3 lots based on genetic lineage through miRNA analysis.

A total of 938 significant protein components were identified through Liquid Chromatography with tandem mass spectrometry (LC-MS/MS) analysis for protein components in exosomes. As a result of analyzing the mutual consistency of all protein components in the exosomes of the three lots by Venn diagram, it was confirmed that 89.6% of the protein components were the same. In addition, in Pearson correlation analysis, it was analyzed that the correlation coefficient between lots was 0.9 or higher (the same if 1). As a result of the ANOVA test analysis, the P value was confirmed to be 0.629 (interpretation that there was a statistically significant difference when it was 0.5 or less), indicating that there was no difference between samples **(Figure 4A)**.

As a result of checking the ratio of LC-MS/MS analysis chromatogram and lipid class, the analysis peak of similar patterns was confirmed in the exosome samples of three lots. It was confirmed that there was no difference between lots as it was commonly detected in exosome samples in the order of PE (Phosphatidylethanolamine), PC (Phosphatidylcholine), and PS (Phosphatidylserine) **(Figure 4B)**.

A profiling analysis was performed based on miRNA to determine whether it has a difference at the level of nucleic acids different from UCBMSC released exosomes (ExoPlus-001, 002 and 003). For comparative experiments group, we used HEK293 (immortalized embryonic kidney cells), Calu3 (lung cancer cells), HKC, MCF7 (breast cancer cells) cells, and DMEM-exo. DMEM-exo is an exosome obtained by culturing in UCBMSCs in commercially available DMEM media. As a result of the analysis, it was confirmed that the miRNA expression pattern between UCBMSC released exosomes (ExoPlus-001~003 shown in red dotted lines) was the most similar. The next experimental group with high genetic similarity to ExoPlus^TM^ was DMEM-exo and UCBMSCs. Other HEK293, Calu3, HKC, and MCF7 were found to have miRNA patterns different from umbilical cord blood stem cell released exosomes **(Figure 4C)**.

In order to confirm the safety of miRNA in UCBMSC released exosomes, the intracellular function of the top eight miRNAs with significant expression in common in three lots was explored. Eight miRNAs were found to be involved in promoting regeneration while protecting various damaged tissues and inhibiting proliferation and metastasis of cancer cells **(Table 3) 15-22**.

### Exosome quality control for GMP manufacturing

For strict quality control as a biopharmaceutical, the quality test items suggested in the guidelines of the KMFDS were set. For a total of 12 items, acceptance criteria and test methods were established that match the characteristics of UCBMSC released exosomes **(Table 4)**. In addition, to ensure the absence of fetal bovine serum (FBS), an alien species used during stem cell culture, in the final exosome raw material, we analyzed the content of FBS throughout the entire process from stem cell culture, conditioned media securing, and exosome isolation/purification in the culture medium. As a result of the analysis, it was confirmed that the FBS content was confirmed during the stem cell culture process, but there was no FBS after the induction of exosome secretion through the cell culture medium washout process of 3 times. (**Suppl. Figure 2**). These results show that there are no alien species substances in the final exosome raw material. Based on these contents, there have been no approved cases of drugs containing exosomes as the main ingredient, so the quality test items and standards for exosomes will be confirmed while discussing with the KMFDS in the future.

### Efficacy and mechanism of UCBMSC released exosomes

Various experiments were conducted to confirm the treatment effect of the UCBMSCs MCB cell line and exosomes isolation and purification process on inflammation suppression and tissue regeneration. There are various diseases that require inflammation suppression and tissue regeneration, but this paper verified the effectiveness of exosomes on wound.

**Figure 5:**
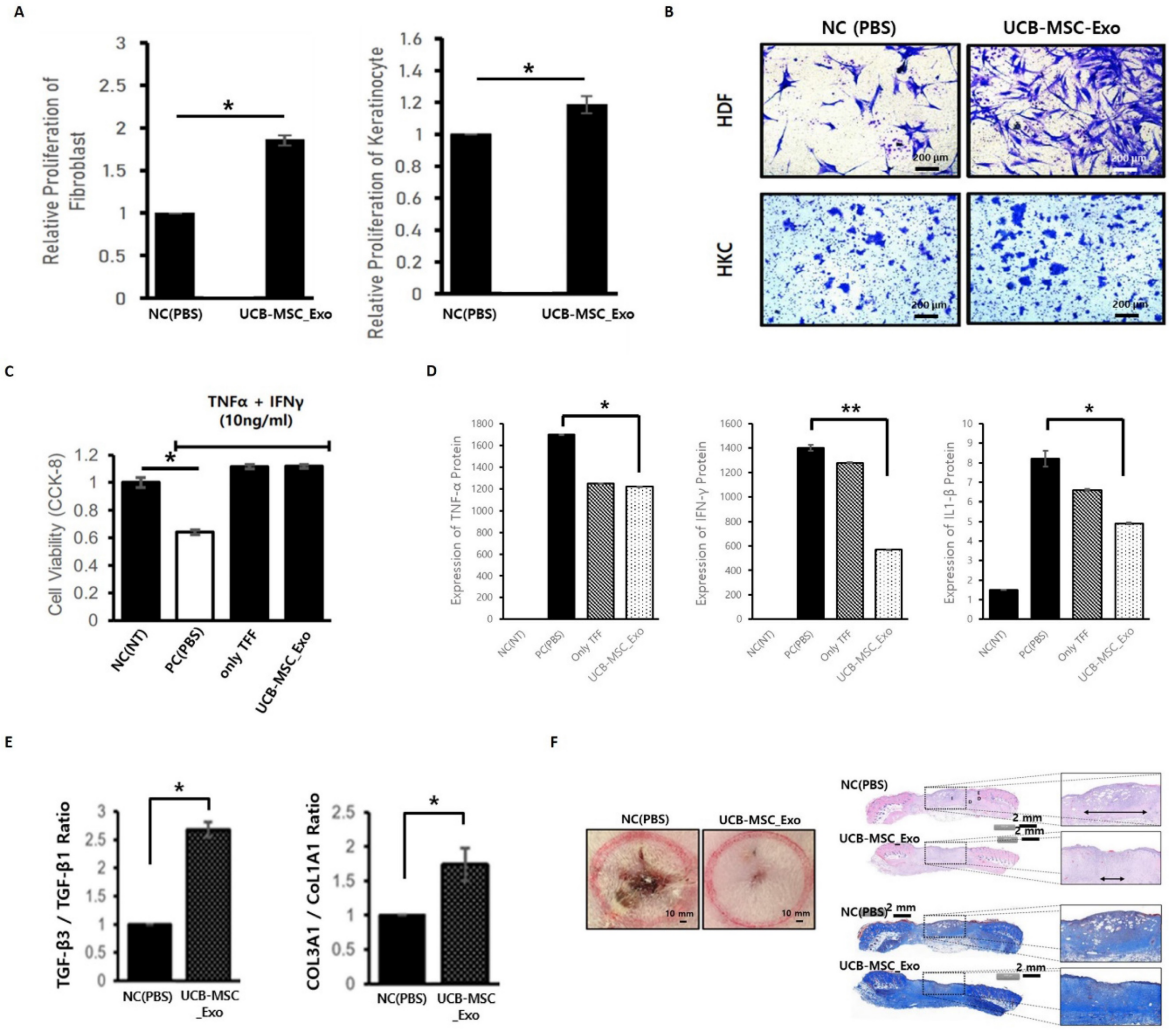
Verification of inflammation inhibition and tissue regeneration efficacy of UCBMSC released exosomes. **(A)** In-cell non-toxic confirmation and inducing cell proliferation when exosomes are treated during skin cell culture. **(B)** Effect of exosomes to promote skin cell movement. **(C)** Effect of inhibiting skin cell death by exosomes in an inflammatory environment (NC (NT), non-treatment; PC (PBS), PBS treatment; only TFF, exosomes treatment using first isolation step' exosomes; UCB-MSC-Exo, exosome treatment using one's completed the isolation/purification process). **(D)** Confirmation of inhibition of inflammatory-inducing substance secretion in skin cells by exosome treatment in an inflammatory environment (NC (NT), non-treatment; PC (PBS), PBS treatment; only TFF, exosomes treatment using first isolation step' exosomes; UCB-MSC-Exo, exosome treatment using one's completed the isolation/purification process). **(E)** Analysis of the expression rate of genes related to scar occurrence. The higher the expression of TFG-β3 and COL3A1, the more stable tissue regeneration is possible without scarring. **(F)** Verification of tissue regeneration efficacy of UCBMSC releasing exosomes using wound disease animal models. On the 10th day after wound induction and drug treatment, the appearance of the affected area (left), and the degree of regeneration through tissue staining of the affected area (right, H&E staining and Masson's Trichrome Staining). Data are shown as mean SD; *P<0.05, **P<0.01, significant compared to control and experimental groups.

To determine whether exosomes themselves act as toxin on skin cells, both skin cells were found to maintain stable cell survival without cell death when exosomes were treated during HDF and HKC. In addition, as a result of checking the degree of growth according to the proliferation of skin cells to check the effect of exosomes on the growth itself of skin cells, it was confirmed that the growth of two skin cells increased in the experimental group treated with exosomes **(Figure 5A)**. In order for the regeneration of the wound (deprivation of skin tissue) to proceed quickly, cells around the wound need to move quickly and form normal tissue. Cell mobility confirmation tests were conducted to confirm the effect of exosomes on these wound healing mechanisms, and the experiment confirmed that the movement of skin cells was actively induced in the experimental group treated with exosomes **(Figure 5B)**.

UCBMSC released exosomes were treated after inducing the inflammatory environment to determine whether exosomes not only protect skin cells from the inflammatory environment but also inhibit the inflammatory process itself. As a result of the experiment, the death of skin cells is suppressed in the exosome treatment experimental group, showing that exosomes protect cells from harmful environments **(Figure 5C)**. After exosome treatment, the amount of inflammation-inducing factors (TNF-α, IFN-γ and IL-β1) secreted by skin cells in an inflammatory environment was measured **(Figure 5D)**. As a result of the experiment, it was confirmed that exosomes have the effect of protecting cells and inhibiting the inflammatory response in an inflammatory environment.

Scarring remains a side effect if regeneration progresses more than necessary in the process of healing after damage to skin tissue. In order to confirm whether exosomes stably induce regeneration without side effects in the process of healing the wound, the expression of genes involved in scar development was confirmed. As a result of the analysis, it was possible to confirm the gene expression pattern of the mechanism of inhibiting tissue regeneration and scarring in the exosome treatment group **(Figure 5E)**.

In order to confirm that the inflammation inhibition and tissue regeneration efficacy of UCBMSC released exosomes identified under various *in vitro* experimental conditions using skin cells, the effectiveness in the body was verified using the wound disease animal model. After inducing a wound using Punch on Rat's back skin, it was compared with the experimental group treated with exosomes on the wound and the control group treated with PBS. As a result of the experiment, it was confirmed that wound healing progressed significantly faster in the experimental group treated with exosomes **(Figure 5F)**.

**Figure 6:**
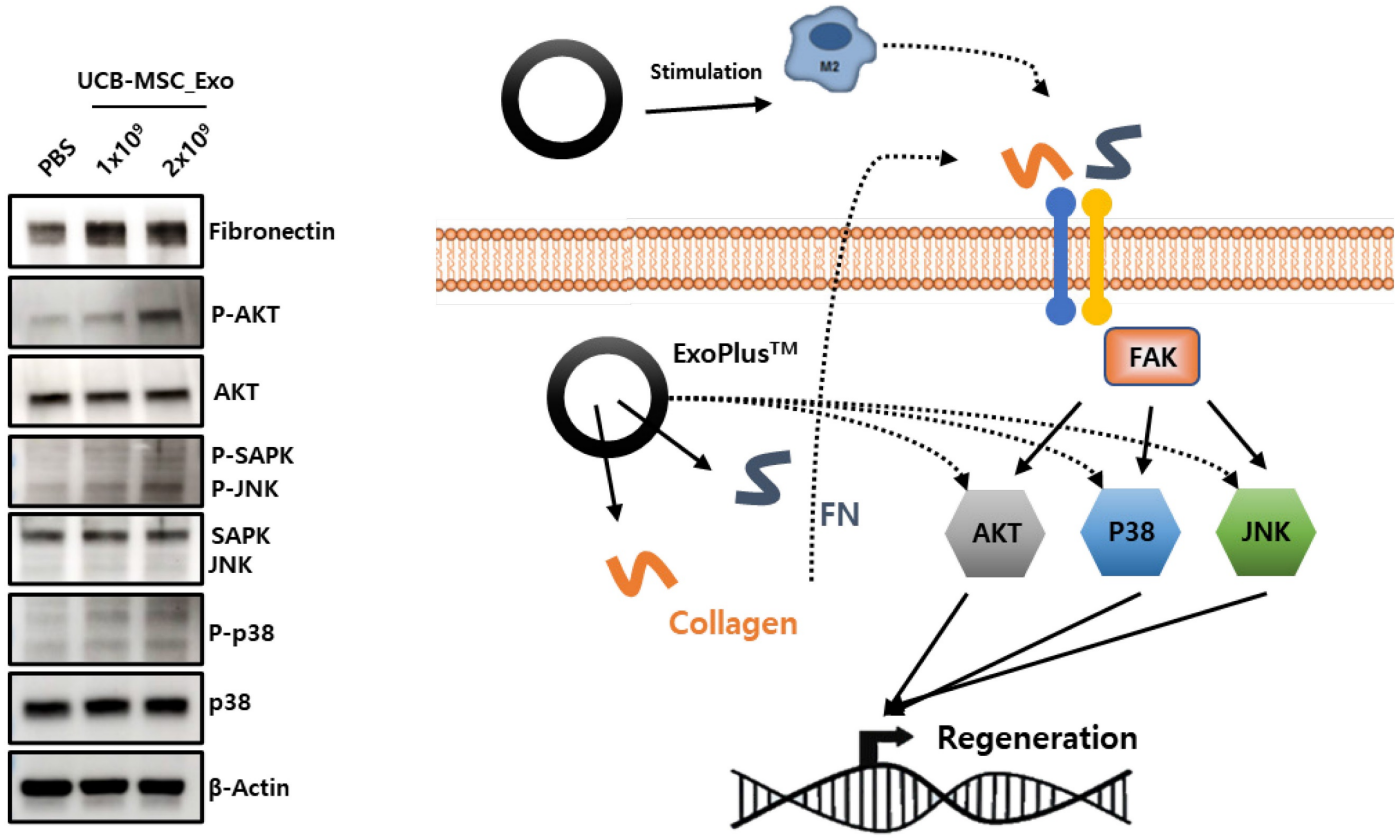
The verification of MoA by analysis of protein expression (left) and schematic diagram (right) of exosome's wound healing effect.

To identify the mechanism by which UCBMSC released exosomes are involved in skin tissue regeneration, it was confirmed that collagen and fibronectin inside the exosome activate sub-materials of FAK signal pathway to promote gene expression involved in tissue regeneration. Through this analysis, this shows the possibility that exosomes can be used as drugs for wound treatments that require tissue regeneration efficacy **(Figure 6)**.

## Discussion

In order to use UCBMSC released exosomes as biopharmaceutical drugs, three guidelines published by the KMFDS were referenced in this study. First, based on the contents of "Guideline on Eligibility Deterrence for Donors of Cell Therapy Products," the safety of the origin material for drugs using human cell-derived materials was verified. In addition to cord blood, the blood of the mother who donated UCB was also collected and tested for items within the guidelines to confirm the suitability of the donor. This can be interpreted as double-checking the safety of the source material for exosome production. Secondly, "Guideline for Cell Bank Evaluation of Cell Therapy Products" was referred to in establishing a standard for establishment and managing a MCB using stem cells isolated from UCB. The guidelines suggest principles for understanding cell bank characteristics and proper management of a series of processes for manufacturing high-quality cell therapy drugs and effective quality control, which can be applied to the establishment of a MCB for stem cell released exosomes.

The guidelines suggest principles for understanding cell bank characteristics and proper management of a series of processes for manufacturing high-quality cell therapy drugs and effective quality control, which can be applied to the establishment of a MCB for stem cell released exosomes. Testing and confirmation of suitability for various items in the previous two guidelines serve as a criterion for determining whether work using the cells is possible within the drug manufacturing GMP facility. Cells identified as meeting both guidelines' quality control items are built as MCBs within GMP and then used for cell culture, securing an exosome-containing culture medium.

Exosomes produced at GMP facilities were set up by referring to the "Guideline on Quality, Non-clinical and Clinical Assessment of Extracellular Vesicles Therapy Products" published by the KMFDS. In the case of this guideline, it is the first published worldwide and mentions the analysis of basic characteristics of exosomes and non-clinical trials that must be met when developing drugs using exosomes. In this study, the basic characteristics of exosomes were analyzed according to the contents of the guidelines and set as a quality control test item, and non-clinical tests will be conducted in the future to confirm safety. In this study, an exosome production process and quality management system for pharmaceuticals that can be manufactured at GMP facilities was established by referring to three related guidelines published by KMFDS.

The dictionary meaning of Wound means wounds and injuries caused by knives or guns, and broadly, it means that skin tissue is broken or defective due to body damage caused by external forces. Many reports have been published to verify the function of wound healing using exosomes released from stem cells of various tissues **23**. The possibility of wound healing therapy of exosomes secreted from various cells such as fat **24**, bone marrow **25**, UCB **26**, and induced pluripotent stem cells **27** was confirmed.

There are not many papers verifying the efficacy of UCBMSC released exosomes with technical hurdles for isolation/purifying and mass production. A report has only been published that targets diabetes **28**, acute liver injury **29**, and wound healing **30**. According to a study by Zhang et al., it was verified that the function of the TGF-β receptor was inhibited by miR-21-5P and miR-125b-5P inside the UCBMSC released exosome, and the exosome for the study was produced as a lab scale **30**. A more in-depth analysis is needed for the various types of miRNAs present in the exosome. In addition to analyzing the types and sub-signals of miRNAs involved in the function of exosomes, it is also necessary to check the absence of miRNAs related to oncogenicity that promote abnormal cell proliferation. The literature has shown that ECM molecules such as collagen and fibronectin stimulate FAK signaling 31, and that FAK signaling in turn promotes downstream AKT, p38, and JNK signaling to promote cell proliferation and migration in liver 32, lung 33, and skin 34 tissues. This supports our findings that ECM molecules in cord blood stem cell exosomes stimulate FAK and downstream signaling to promote regeneration of damaged skin tissue.

The UCBMSC released exosomes secured through this study not only have the effect of suppressing inflammation and tissue regeneration inherent in UCBMSCs, but also have been secured through a mass production process for commercial use. Therefore, it can be used as a drug for various diseases that require the treatment mechanism. In this study, the healing effect on wound was first verified. Since then, we are developing an exosome-based drug for core second-degree burns based on wound healing efficacy. In order to effectively treat exosome drugs in burn affected areas, we are considering various types of morphing, not liquid. In addition, various diseases that require inflammation suppression and tissue regeneration mechanisms that can maximize the efficacy of exosomes during intravenous and subcutaneous injections are being explored. Active research on various remodeling and diseases using exosomes will serve as an important foundation for building the unique field of exosome-based biopharmaceuticals in the future.

In this study, a MCB was established by isolating stem cells from UCB, and then using MCB cell lines to secure UCBMSC releasing exosomes. In accordance with the three guidelines presented by KMFDS, the production process was established to enable manufacturing and quality control within the GMP. The exosomes secured in this way have the effect of suppressing inflammation and regenerating tissue, and these characteristics suggest the possibility of using exosomes as a new biomedicine.

## Supplementary Material

Supplementary figure.

## Figures and Tables

**Table 1:** Methods and results of blood inspection items for eligibility determination for donors

Inspection Items	Inspection Method	Conformity	Results
Hepatitis B virus(HBV)	Surface antigen (HBs Ag) test for HBV	Negative	Negative
	Nucleic acid amplification (NAT) test for HBV	Negative	Negative
Hepatitis C virus (HCV)	Surface antigen (HBs Ag) test for HCV	Negative	Negative
	Nucleic acid amplification (NAT) test for HCV	Negative	Negative
Human Immuno- deficiency virus 1 and 2 (HIV 1/2)	Antigen test for HIV 1/2	Negative	Negative
	Nucleic acid amplification (NAT) test for HIV 1/2	Negative	Negative
Syphilis	Non-Treponema test	Negative	Negative
	Treponema test	Negative	Negative
Human T- lymphotropic virus (HTLV)	Antigen test for HTLV	Negative	Negative
Cytomegalovirus (CMV)	Antigen test for CMV	Negative	Negative
	Nucleic acid amplification (NAT) test for CMV	Negative	Negative

**Table 2:** Characterization of Cells for MCB

Inspection Item		Standard	Results
Cells	Identity	Self-standard	Suitable
	Purity	Self-standard	Suitable
	Potency	Self-standard	Suitable
	Stability	Self-standard	Suitable
	Morphology	Self-standard	Suitable
	Growth characteristic	Self-standard	Suitable
Sterility	Bacteria, Fungi	Negative	Negative
	Mycoplasma	Negative	Negative
Adventitious agent	*In vitro* assay	Negative	Negative
	*In vivo* assay	Negative	Negative
	Bovine viruses	Negative	Negative
	Retroviruses	Negative	Negative
	Tests for human viruses^1)^	Negative	Negative

1) HBV, HCV, HIV1/2, HAV, HHV6/7/8, EBV, HTLV1/2, HCMV, Human parvovirus P19, HPV, Human adenovirus)

**Table 3:** Types and functions of miRNAs commonly present in exosomes of 3 lots

Ranking	The type of miRNAs	Function of miRNAs
1	miR-100-5p	Promotion of wound healing on damaged skin 15
2	miR-22-3p	Suppression of cell growth in lung cancer 16
3	miR-181a-5p	Protection of kidney injury in sepsis 17
4	miR-127-3p	Inhibition of cell growth and invasiveness in osteosarcoma 18
5	miR-92a-3p	Enhancing of functional recovery and suppresses apoptosis after spinal cord injury 19
6	miR-21-5p	Promotion of skin wound healing 20
7	Let-7a-5p	Suppression of inflammation in liver injury 21
8	miR-27b-3p	Attenuation of fibrosis in heart 22

**Table 4:** Specification of control of active substance using exosomes

Test item		Acceptance criteria	Method
Morphology		Self-standard	Visual observation
pH		Self-standard	pH meter measurement
Sterility		Negative	The Korean Pharmacopoeia standards
Mycoplasma		Negative	Real-time PCR analysis
Endotoxin		Self-standard	The Korean Pharmacopoeia standards
Adventitious virus		Negative	Guideline method of KMFDS
Confirmation	Size and number of exosomes	Self-standard	Nanoparticles Tracking Analyzer (NTA) analysis
	Exosomes surface and cell organs markers	Self-standard	Flow cytometry analysis
	Effective substance	Self-standard	Flow cytometry analysis
Purity	Protein	Self-standard	ELISA analysis
	Nucleic acid	Not detected	Real-Time PCR analysis
Potency	Related gene expression	Self-standard	Real-Time PCR analysis

**Table 5:** Sequences of primers for real-time PCR

Gene	Primer Sequence (5' to 3')	
	Forward	Reverse
GAPDH	GAAAGCCTGCCGGTGACTAA	AGGAAAAGCATCACCCGGAG
PPAR-γ	AGCCTGCGAAAGCCTTTTGGTG	GCGGCTTCACATTCAGCAAACCTGG
Osteopontin	CGAGGTGATAGTGTGGTTTATGG	GCACCATTCAACTCCTCGCTTTC
Sox9	AGGAAGCTCGCGGACCAGTAC	GGTGGTCCTTCTTGTGCTGCAC
Collagen 1	GATTCCCTGGACCTAAAGGTGC	GCCTCTCCATCTTTGCCAGC
Collagen 3	GTCTGCAAGGAATGCCTGGA	CTTTCCCTGGGACACCATCAG
TGF-β1	ACCTGAACCCGTGTTGCTCT	TTGCTGAGGTATCGCCAGGA
TGF-β3	CTAAGCGGAATGAGCAGAGGATC	TCTCAACAGCCACTCACGCA
Cytochome C	AAGGGAGGCAAGCACAAGACTG	CTCCATCAGTGTATCCTCTCCC
ERP29	GCAGGATGAGTTCAAGCGTCTTG	GCTCCATGTTCAGCTTGTCACC
GOM1	ATCACCACAGGTGAGAGGCTCA	ACTTCCTCTCCAGGTTGGTCTG
NUP153	ACTACCACCTCTGGTTTCGGCT	CCAAATGCTGGACTGGCAGATG

## Abbreviations

KMFDS: the Korean ministry of food and drug safety
EVs: extracellular vesicles
GMP: good manufacturing practice
UCBSCs: umbilical cord blood stem cells
KoNIBP: Korea national institute for bioethics policy
TEM: transmission electron microscope
HDF: human dermal fibroblasts
HKC: human keratinocyte
ATCC: American type culture collection
SDS-PAGE: sodium dodecyl sulfate-polyacrylamide gel electrophoresis
SD: standard deviation
MCB: master cell bank
TFF: tangential flow filtration
SEC: size exclusion chromatography
NTA: nano tracking analysis
LC-MS/MS: liquid chromatography with tandem mass spectrometry
