# Supplementary material for: GMP manufacturing of umbilical cord blood mesenchymal stem cell-released exosomes and verification of wound healing efficacy
Source: Nanotheranostics. 2025 Mar 3;9(2):95–109. doi: 10.7150/ntno.105433 (PMC11980038; doi:10.7150/ntno.105433)
Supplement: Supplementary file 1 — Supplementary figure. [file ntnov09p0095s1.pdf]

Supplementary Figure 1

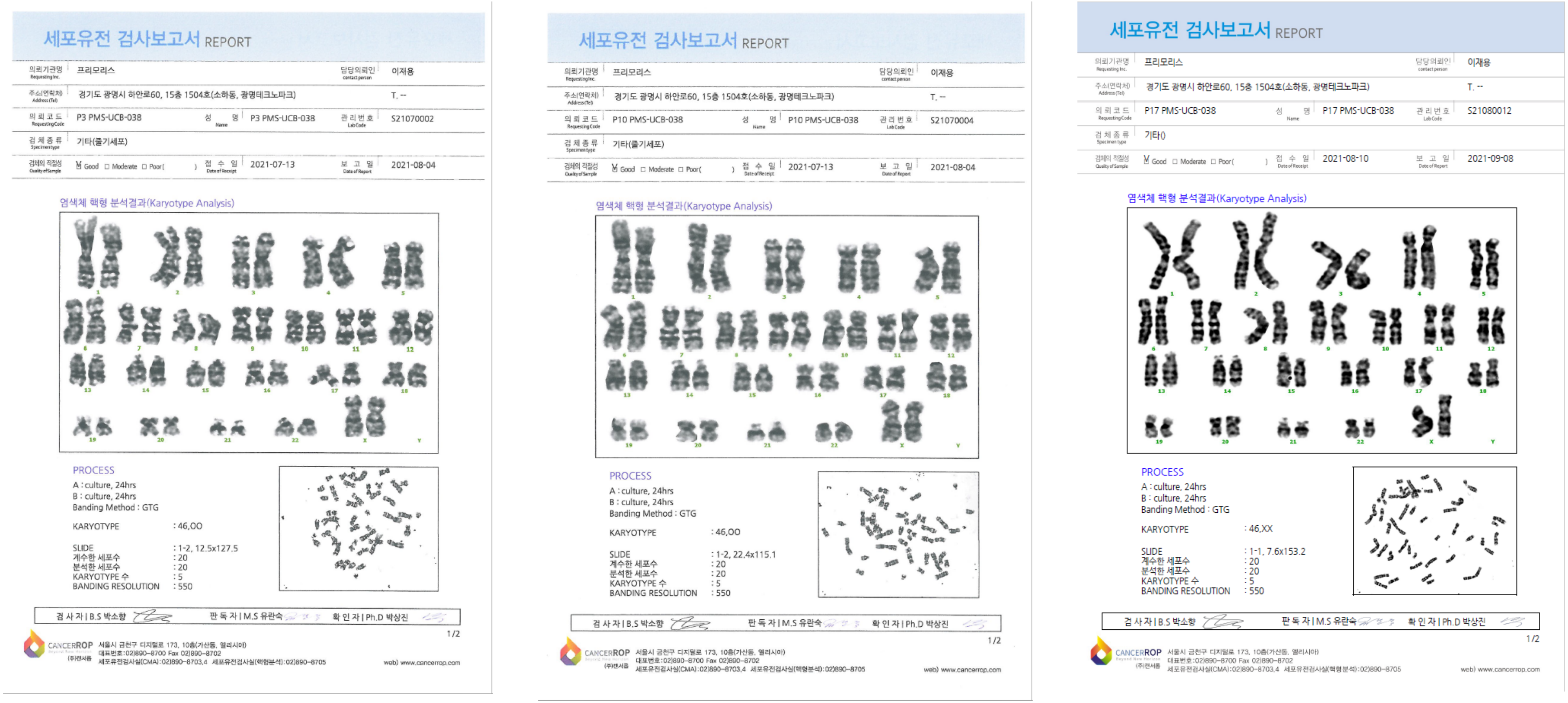

세포유전 검사보고서 REPORT

의뢰기관명 Requesting Inc.	프리모리스	담당의뢰인 contact person	이재용
주소(연락처) Address(Tel)	경기도 광명시 하안로60, 15층 1504호(소하동, 광명테크노파크)		T. --
의 뢰 코 드 Requesting Code	P3 PMS-UCB-038	성 명 Name	P3 PMS-UCB-038
관 리 번 호 Lab Code	S21070002		
검 체 종 류 Specimen type	기타(줄기세포)		
검체의 적절성 Quality of Sample	V Good ☐ Moderate ☐ Poor( )	접 수 일 Date of Receipt	2021-07-13
보 고 일 Date of Report	2021-08-04		

염색체 핵형 분석결과(Karyotype Analysis)

PROCESS
A : culture, 24hrs
B : culture, 24hrs
Banding Method : GTG

KARYOTYPE : 46,OO

SLIDE : 1-2, 12.5x127.5
계수한 세포수 : 20
분석한 세포수 : 20
KARYOTYPE 수 : 5
BANDING RESOLUTION : 550

검 사 자 I B.S 박소향 판 독 자 I M.S 유란숙 확 인 자 I Ph.D 박상진

1/2

CANCERROP (주)캔서롭 서울시 금천구 디지털로 173, 10층(가산동, 엘리시아) 대표번호:02)890-8700 Fax 02)890-8702 세포유전검사실(CMA):02)890-8703,4 세포유전검사실(핵형분석):02)890-8705 web) www.cancerrop.com

세포유전 검사보고서 REPORT

의뢰기관명 Requesting Inc.	프리모리스	담당의뢰인 contact person	이재용
주소(연락처) Address(Tel)	경기도 광명시 하안로60, 15층 1504호(소하동, 광명테크노파크)		T. --
의 뢰 코 드 Requesting Code	P10 PMS-UCB-038	성 명 Name	P10 PMS-UCB-038
관 리 번 호 Lab Code	S21070004		
검 체 종 류 Specimen type	기타(줄기세포)		
검체의 적절성 Quality of Sample	V Good ☐ Moderate ☐ Poor( )	접 수 일 Date of Receipt	2021-07-13
보 고 일 Date of Report	2021-08-04		

염색체 핵형 분석결과(Karyotype Analysis)

PROCESS
A : culture, 24hrs
B : culture, 24hrs
Banding Method : GTG

KARYOTYPE : 46,OO

SLIDE : 1-2, 22.4x115.1
계수한 세포수 : 20
분석한 세포수 : 20
KARYOTYPE 수 : 5
BANDING RESOLUTION : 550

검 사 자 I B.S 박소향 판 독 자 I M.S 유란숙 확 인 자 I Ph.D 박상진

1/2

CANCERROP (주)캔서롭 서울시 금천구 디지털로 173, 10층(가산동, 엘리시아) 대표번호:02)890-8700 Fax 02)890-8702 세포유전검사실(CMA):02)890-8703,4 세포유전검사실(핵형분석):02)890-8705 web) www.cancerrop.com

세포유전 검사보고서 REPORT

의뢰기관명 Requesting Inc.	프리모리스	담당의뢰인 contact person	이재용
주소(연락처) Address(Tel)	경기도 광명시 하안로60, 15층 1504호(소하동, 광명테크노파크)		T. --
의 뢰 코 드 Requesting Code	P17 PMS-UCB-038	성 명 Name	P17 PMS-UCB-038
관 리 번 호 Lab Code	S21080012		
검 체 종 류 Specimen type	기타()		
검체의 적절성 Quality of Sample	V Good ☐ Moderate ☐ Poor( )	접 수 일 Date of Receipt	2021-08-10
보 고 일 Date of Report	2021-09-08		

염색체 핵형 분석결과(Karyotype Analysis)

PROCESS
A : culture, 24hrs
B : culture, 24hrs
Banding Method : GTG

KARYOTYPE : 46,XX

SLIDE : 1-1, 7.6x153.2
계수한 세포수 : 20
분석한 세포수 : 20
KARYOTYPE 수 : 5
BANDING RESOLUTION : 550

검 사 자 I B.S 박소향 판 독 자 I M.S 유란숙 확 인 자 I Ph.D 박상진

1/2

CANCERROP (주)캔서롭 서울시 금천구 디지털로 173, 10층(가산동, 엘리시아) 대표번호:02)890-8700 Fax 02)890-8702 세포유전검사실(CMA):02)890-8703,4 세포유전검사실(핵형분석):02)890-8705 web) www.cancerrop.com

Karyotyping results for each lineage (P3, P10 and P17) to confirm the genetic stability of the umbilical cord blood mesenchymal stem cell lines selected for the establishment of the master cell bank.

Supplementary Figure 2

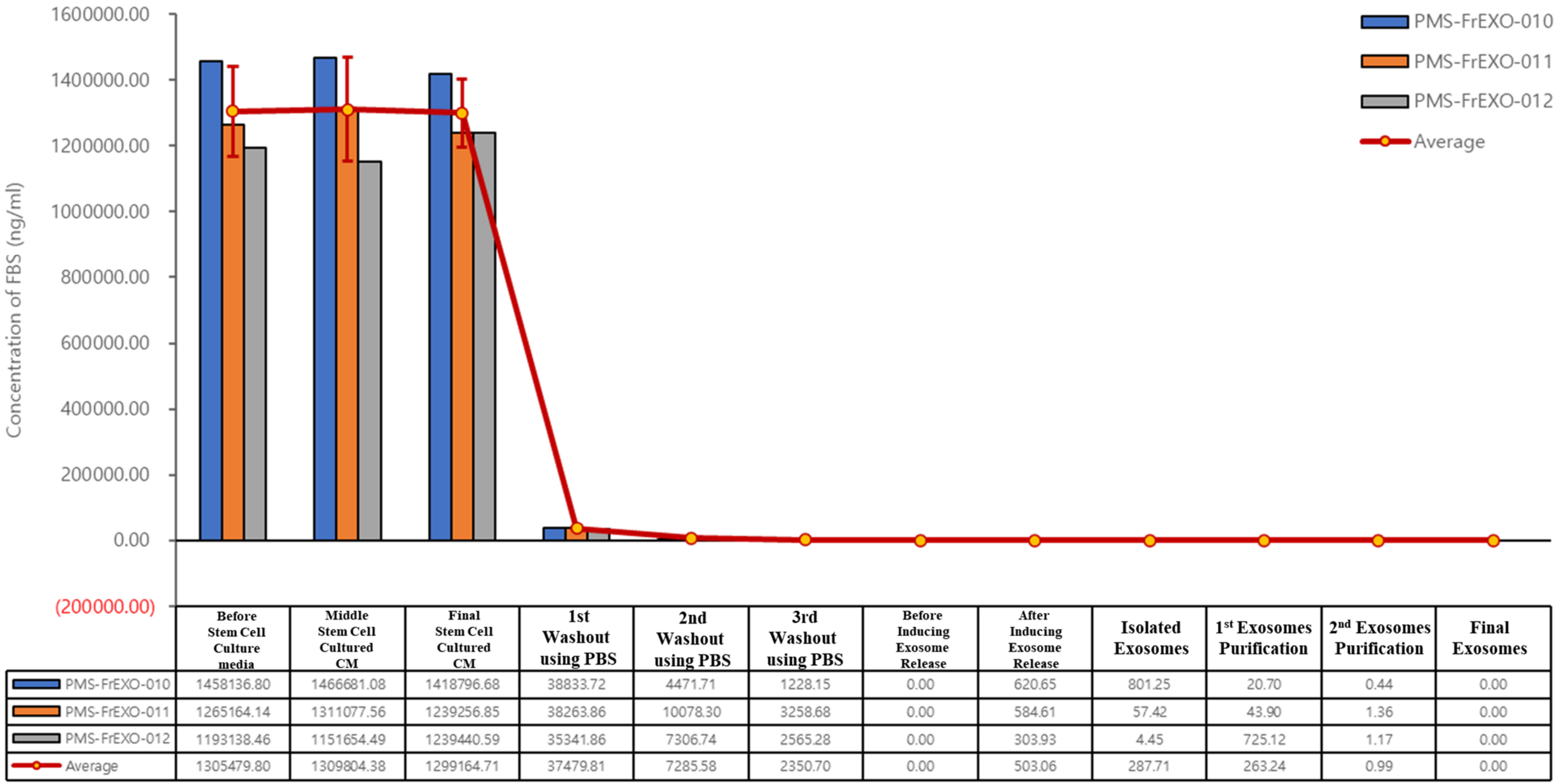

	Before Stem Cell Culture media	Middle Stem Cell Cultured CM	Final Stem Cell Cultured CM	1st Washout using PBS	2nd Washout using PBS	3rd Washout using PBS	Before Inducing Exosome Release	After Inducing Exosome Release	Isolated Exosomes	1st Exosomes Purification	2nd Exosomes Purification	Final Exosomes
PMS-FrEXO-010	1458136.80	1466681.08	1418796.68	38833.72	4471.71	1228.15	0.00	620.65	801.25	20.70	0.44	0.00
PMS-FrEXO-011	1265164.14	1311077.56	1239256.85	38263.86	10078.30	3258.68	0.00	584.61	57.42	43.90	1.36	0.00
PMS-FrEXO-012	1193138.46	1151654.49	1239440.59	35341.86	7306.74	2565.28	0.00	303.93	4.45	725.12	1.17	0.00
Average	1305479.80	1309804.38	1299164.71	37479.81	7285.58	2350.70	0.00	503.06	287.71	263.24	0.99	0.00

Measure FBS content in samples at every step of the process, from cord blood stem cell culture media to final exosome isolation/purification. The data shows that FBS, which was contained during the USBMSCs culture, is no longer detectable after three PBS washouts. CM; conditioned media

Supplementary Figure 3

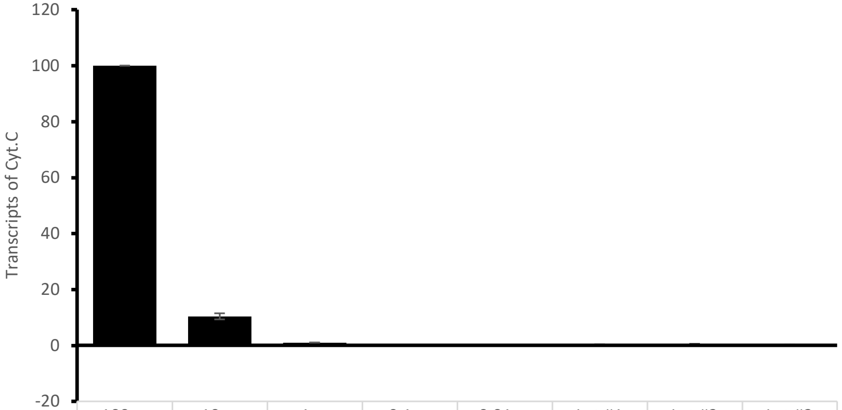

	100ng	10ng	1ng	0.1ng	0.01ng	Lot #1	Lot #2	Lot #3
■ Cyt.C	100	10.3712148	0.98171441	0.12694059	0.07098137	0.29914512	0.53036348	0.28936844
CV	0.00%	10.34%	8.13%	2.41%	12.03%	6.27%	7.19%	3.13%

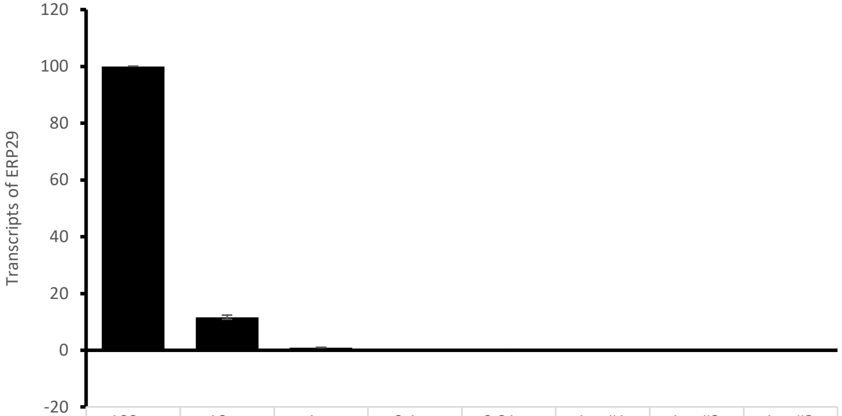

	100ng	10ng	1ng	0.1ng	0.01ng	Lot #1	Lot #2	Lot #3
■ ERP29	100	11.6285708	0.98751274	0.07716491	0.01188291	0	0	0
CV	0.00%	6.27%	2.39%	3.83%	23.88%	0.00%	0.00%	0.00%

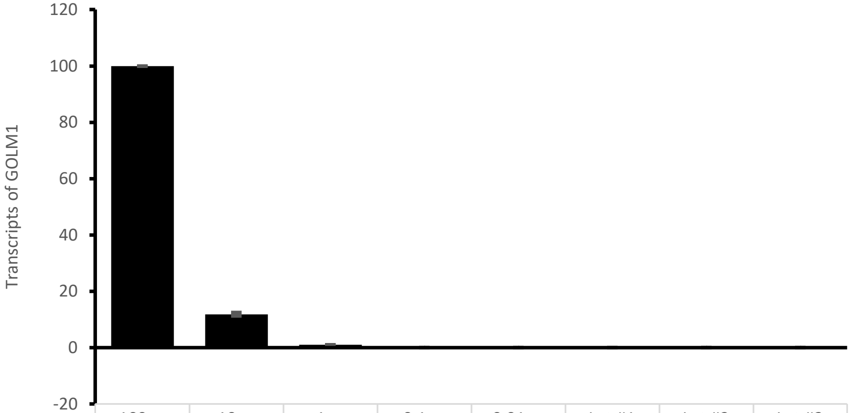

	100ng	10ng	1ng	0.1ng	0.01ng	Lot #1	Lot #2	Lot #3
■ GOLM1	100	11.8760386	1.06828571	0.08250014	0.01534621	0.00074759	0.00138855	0.00076858
CV	0.00%	5.35%	1.50%	1.65%	10.32%	3.18%	17.44%	20.82%

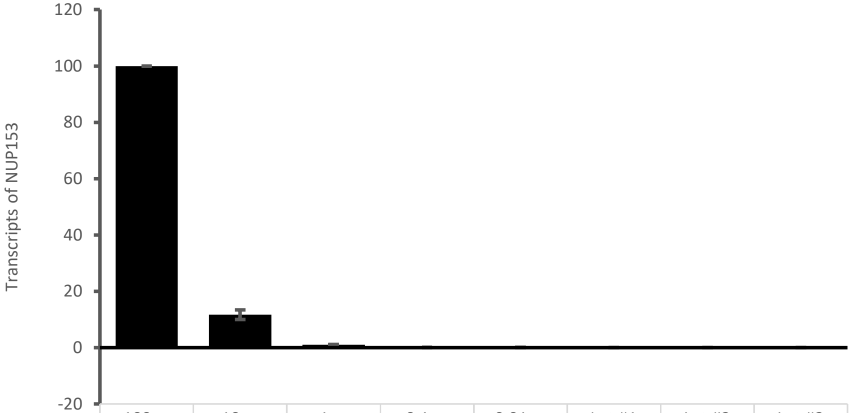

	100ng	10ng	1ng	0.1ng	0.01ng	Lot #1	Lot #2	Lot #3
■ NUP153	100	11.7161705	1.02623986	0.07976341	0.01406823	0	0	0
CV	0.00%	14.73%	8.09%	14.70%	43.93%	0.00%	0.00%	0.00%

RT-PCR analysis of genes specifically expressed in mitochondria(Cyt.C), endoplasmic reticulum(ERP29), golgi(GOLM1), and nucleus(NUP153) to determine if the final exosome materials contains other cell organelles besides exosomes. Confirmed the expression of each gene with three lots of exosome material using stem cell cDNA for each concentration as a positive control, confirming that the exosome material obtained does not contain cell organelles.
